# Delayed Neurologic Response to Dabrafenib and Trametinib in the Case of Mixed Histiocytosis (LCH/ECD): Case Report and Literature Review

**DOI:** 10.3390/reports9010018

**Published:** 2026-01-07

**Authors:** Shinsaku Imashuku, Miyako Kobayashi, Takashi Miyoshi, Naoyuki Anzai

**Affiliations:** 1 Division of Hematology, Uji-Tokushukai Medical Center, Uji 611-0041, Kyoto, Japan; saku3939@kuhp.kyoto-u.ac.jp (T.M.); anzai@ujitoku.or.jp (N.A.); 2 Department of Laboratory Medicine, Uji-Tokushukai Medical Center, Uji 611-0041, Kyoto, Japan; 3 Department of Internal Medicine, Uji-Tokushukai Medical Center, Uji 611-0041, Kyoto, Japan; miyak.3330@gmail.com

**Keywords:** Langerhans cell histiocytosis, Erdheim–Chester disease, mixed histiocytosis, dabrafenib, trametinib, neurological symptoms

## Abstract

**Background and Clinical Significance**: Histiocytosis encompasses Langerhans cell histiocytosis (LCH) and non-LCH, such as Erdheim–Chester disease (ECD). ECD or a mixed type of histiocytosis (LCH/ECD) may initially involve the central nervous system (CNS), resulting in a delayed diagnosis. More recently, dabrafenib and trametinib (Dab/Tra regimen) have become available in its treatment. **Case Presentation**: A 46-year-old woman with CNS involvement of mixed histiocytosis (BRAF V600E-positive LCH/ECD) was treated with combination therapy using a Dab/Tra regimen. At initial presentation, she exhibited central diabetes insipidus, dysarthria, and gait disturbance with mild spasticity and ataxia, requiring walking assistance even for short distances. The interval from the onset of central neurological symptoms to diagnosis of mixed histiocytosis was 4 years. The introduction of targeted therapy was 2 years later. After seven months of Dab/Tra therapy, partial neurological improvement was observed, as reflected by a decrease in the SARA score from 21/40 to 13/40 and the ICARS score from 33/100 to 28/100. However, further neurological recovery remained significantly delayed. **Conclusions**: We suspect that the limited improvement may be attributable to the delayed initiation of targeted therapy, in contrast to the more rapid and pronounced responses reported in cases where treatment was started earlier.

## 1. Introduction and Clinical Significance

Histiocytic neoplasms such as Langerhans cell histiocytosis (LCH) and Erdheim–Chester disease (ECD) are characterized by various mutations in the mitogen-activated protein kinase (MAPK) pathway, including *BRAF V600E*, *MAP2K1*, and others [1]. Cases of mixed histiocytosis—defined as the coexistence of both LCH and ECD in the same patient—have also been documented [2,3]. For these disorders, targeted therapies using BRAF inhibitors (e.g., vemurafenib or dabrafenib) and/or MEK inhibitors (e.g., trametinib or cobimetinib) have demonstrated clinical efficacy. Treatment regimens may involve monotherapy with a BRAF or MEK inhibitor, or a combination of both agents [4]. Central nervous system (CNS) involvement—either as tumorous or degenerative lesions—occurs in approximately 10–25% of LCH patients and 30–40% of those with ECD [5]. In CNS-ECD, characteristic involvement of the dentate nucleus and pons results in a pyramido-cerebellar syndrome, with clinical symptoms including gait ataxia with hyperreflexia, dysarthria, chorea, and others. Notably, up to 33% of patients with CNS-ECD succumb to the disease or its complications [6]. Despite growing clinical experience, the literature remains inconclusive regarding the optimal targeted agent(s) and the expected time course for neurologic improvement. **Clinical significance**: Historically, vemurafenib was the first BRAF inhibitor used in CNS-ECD cases [7,8,9,10,11,12,13]. More recently, dabrafenib, either alone or in combination with MEK inhibitors, has also been employed [14,15]. In mixed histiocytosis, treatment strategies are designed to address both ECD and LCH components [5]. Roeser et al. reported a dramatic neurologic response to MEK inhibitor therapy in the case of mixed histiocytosis [16]. Here, we report the therapeutic outcome in an ataxic patient with mixed histiocytosis (ECD/LCH), treated with a combination of dabrafenib and trametinib (Dab/Tra), a regimen now approved for use in Japan.

## 2. Case Presentation

### 2.1. Delayed Diagnosis of CNS Disease Followed by Chemotherapy

**Figure 1:**
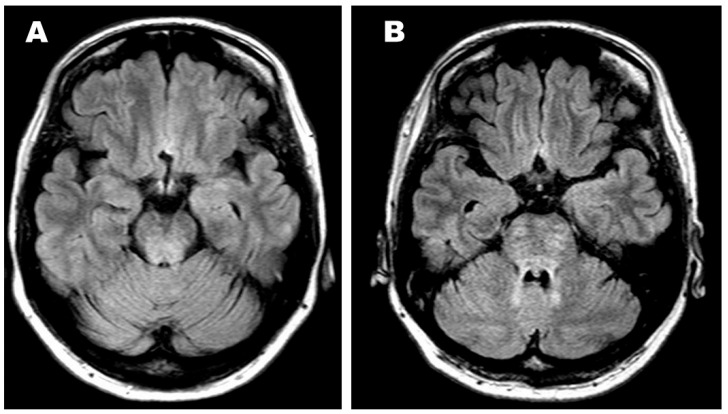
MRI shows a pons lesion. (**A**) At the diagnosis, when histiocytosis was not suspected. (**B**) After 7 months of Dab/Tra therapy for mixed histiocytosis (LCH/ECD).

**Figure 2:**
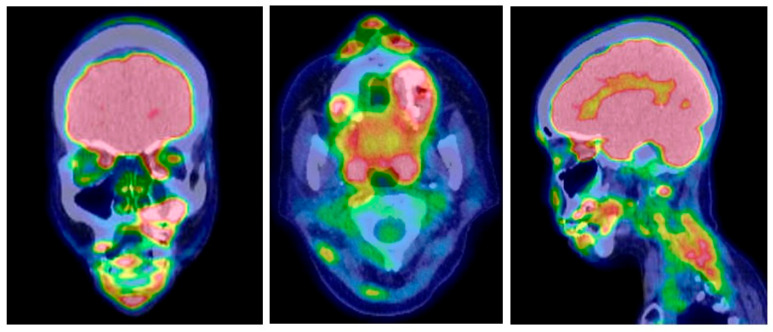
PET/CT findings of the maxillary and mandibular bone lesions at pre-targeted therapy, showing left maxilla and mandible involvement.

A 46-year-old woman developed central diabetes insipidus and gait disturbance, accompanied by mild spasticity and ataxia. This case was previously described by Miyoshi et al. [3]. The patient was unable to walk even with the assistance of a walker and could not maintain a stable sitting position. She had dysarthria and nystagmus, but no swallowing difficulty. She remained alert and oriented, with intact cranial nerves. She had no signs of memory loss or impaired concentration. Brain MRI (T2-weighted imaging) revealed a hyperintense lesion in the pons, resembling chronic lymphocytic inflammation with pontine perivascular enhancement responsive to steroids (CLIPPERS) [17,18] (Figure 1A). Although she initially visited a neurology clinic, no definitive diagnosis was made. Two years later, at age 48, multiple osteolytic lesions, involving the bilateral maxillae, mandible, and femur, were identified. A biopsy from the left maxilla was diagnosed as LCH when no specific therapy was given; however, at age 50, another biopsy of the left femur with re-review of the previous pathological preparations confirmed the diagnosis of mixed histiocytosis (ECD/LCH) harboring the *BRAF V600E* mutation. Thus, in this patient, it took four years from the onset of neurological symptoms to the diagnosis of mixed histiocytosis and the initiation of systemic therapy [3]. Initial treatment with five courses of cladribine (0.1 mg/kg/day for 5 days per course) did not yield noticeable improvement. Off-label treatment with the MEK inhibitor trametinib (0.5 mg/day) was given for six months but was similarly ineffective. Subsequently, she received a “Special C” regimen—vinblastine, methotrexate, 6-mercaptopurine, and prednisolone [19]—without clinical benefit for CNS lesion or the craniofacial bone lesions in the mixed histiocytosis (LCH/ECD). At this point, the neurological symptoms were unchanged. PET/CT demonstrated complete metabolic remission (CMR; Deauville score [DS] 1–2) in the bilateral long bone lesions, whereas partial remission (PR; DS 4–5) persisted in the maxillomandibular bone lesions (Figure 2, Table 1).

### 2.2. Evaluation of Targeted Treatment

Doses of Dab/Tra gradually increased from Dab (50 mg/day)/Tra (0.5 mg/day) to Dab (200 mg/day)/Tra (1.5 mg/day) over 4 months, with concerns for drug toxicity; one major reason was that she lived far away and was only able to visit our hospital every 1 to 2 months. Eventually, the maximum dose was administered at Dab (250 mg/day)/Tra (2.0 mg/day). Clinical response was evaluated every 3–6 months using neurological scales (SARA and ICARS), while imaging studies (brain MRI and PET-CT) were assessed every 6–12 months. Adverse events (AEs) and their severity were recorded according to the Common Terminology Criteria for Adverse Events (CTCAEs).

### 2.3. Outcome of Targeted Treatment

**Figure 3:**
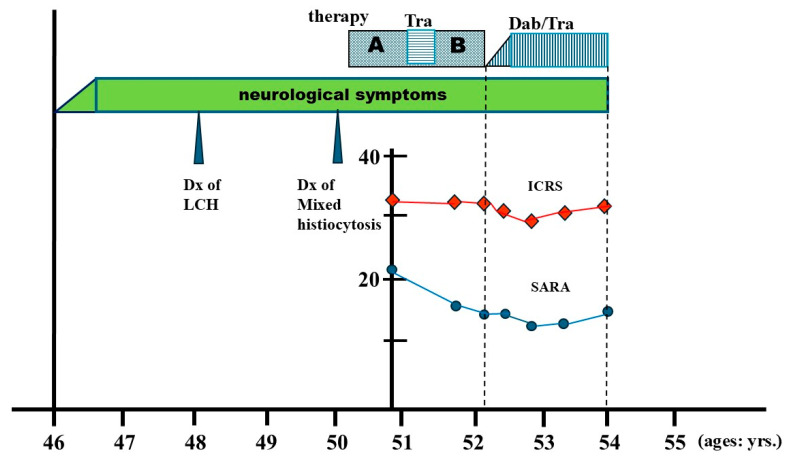
The clinical course of the patient.

After the approval of the dabrafenib and trametinib combination (Dab/Tra) in Japan, targeted therapy was initiated 6 years from the initial CNS symptoms, and 2 years after the diagnosis of mixed histiocytosis. At pre-targeted treatment, the left maxillary/mandibular lesions also remained in PR on PET/CT (Figure 2). As mentioned above, treatment began with Dab (50 mg/day)/Tra (0.5 mg/day), then increased up to Dab (250 mg/day)/Tra (2 mg/day). After 7 months of treatment, the SARA score improved to 13/40, and the ICARS score improved slightly to 28/100. At that point, an objective partial response—mild improvements in dysarthria and assisted ambulation for short distances—was obtained, in association with an improved pons lesion (Figure 1B). However, after 18 months, as summarized in a timeline of the patient’s clinical course (Figure 3), her SARA was 14/30, but ICARS score 29/100, and at 21 months, her SARA score 17/40 while ICARS score 31/100, indicating that there were some contradictory results between SARA and ICARS scores. Clinically, her ataxia, spastic gait, and dysarthria persisted even after 21 months of Dab/Tra treatment, as stated below.

Neurological symptoms developed at the age of 46. Though she was diagnosed with LCH at the age of 48, two years later, mixed histiocytosis (LCH/ECD) was confirmed. Treatment was (A) cladribine, (B) Special C regimen. In between, off-label Tra (trametinib) was given. After the Special C regimen, the Dab/Tra regimen was initiated. The ICARS score (red diamond) and SARA score (blue circle) were plotted during the cladribine treatment. As noted, no significant improvement was observed by the Dab/Tra treatment.

After 21 months of treatment with Dab/Tra, her ataxia, spastic gait, and dysarthria persisted. She lived alone, as both of her daughters were married and lived far away. At home, she ambulated by holding onto handrails and was able to manage toileting and bathing independently. When going out, she used a wheelchair for short distances and was usually driven by others for longer travel. She took her three daily meals at her mother’s nearby house.

## 3. Discussion

As summarized in Table 2, previous reports on CNS-ECD cases treated with vemurafenib are mostly fragmentary, lacking detailed information on drug dosage, treatment duration, clinical response, and long-term outcomes [7,8,9,10,11,12,13]. In these reports, the interval from CNS symptom onset to diagnosis ranged from 2 to 9 years [7,13]. Vemurafenib was administered for durations ranging from 1 to 52 months [7,8,10,11,12,13], and responses were observed as early as 1–4 months after treatment initiation [8,13]. Among the nine reported cases, four showed partial to significant neurological improvement [7,8,10,12]. As for long-term outcomes, Pan et al. reported a disease-free state after three years of treatment [8], and Fernández-Eulate et al. noted the absence of disease progression along with the patient’s ability to walk short distances unassisted [12]. In three cases of 2–9 years’ delay in diagnosis [13], clinical improvement was observed within 2–4 months of treatment, but long-term follow-up data were unavailable.

In contrast, the combination of Dab/Tra was hypothesized to provide more rapid neurological improvement owing to dabrafenib’s superior CNS penetration compared with vemurafenib [20]. In glioma studies, the Dab/Tra combination was noted to penetrate the CNS barrier effectively [21,22]. In pediatric histiocytosis, the following long-term data on the safety and optimal duration of Dab/Tra treatment are available: sustained clinical responses were obtained in 15 of 16 (94%) children with recurrent or refractory LCH and other histiocytic disorders, with a median treatment duration of 4.3 years [23]. However, clinical data on Dab or Dab/Tra therapy in adults remain limited. Elkouzi et al. described a 55-year-old man with ataxia treated with Dab monotherapy, but treatment details and outcomes were not provided [24]. Bhatia et al. reviewed eleven adult cases of ECD or mixed histiocytosis (ECD/LCH) treated with Dab alone [25], including two patients with ataxia and dysarthria who showed partial and complete metabolic responses after 4 and 11 months of therapy, respectively. However, the timing of neurological symptom improvement was not specified. Yuen et al. reported a 44-year-old man with extensive CNS-ECD who began Dab treatment seven weeks after symptom onset, following four weeks of steroid therapy. After three months, clinical and radiological improvements were evident, although Dab dosing was not reported [14]. Al Bayati et al. described a 44-year-old woman with brainstem ECD involvement who was started on Dab/Tra two years after symptom onset. Her dysarthria and gait disturbance significantly improved within four months, and by ten months, only minor neurological deficits remained [15]. Their patient was started on Tra (2 mg/day) and Dab (150 mg/day to 300 mg/day); however, the patient needed multiple dose adjustments of Dab/Tra due to toxicity. Her final regimen was Dab (150 mg/day) and Tra (1 mg/day).

In contrast to these earlier cases, our patient began Dab/Tra therapy six years after the onset of neurological symptoms (Table 3). Partial and transient improvement in neurological symptoms was observed after seven months of treatment; however, further improvement was notably delayed. In addition, the response in the maxillo-mandibular bone lesions was also delayed, even with the Dab/Tra regimen (Table 3). We wondered whether our case of mixed histiocytosis showed any unfavorable predictors. According to Pegorano et al., the main independent predictors of poor survival in the mixed histiocytosis were older age at diagnosis, associated hematologic conditions, and treatment failure [26]. We also suspect that her delay in improving CNS lesions may be attributable to the late initiation of targeted therapy, compared to the earlier intervention reported in the literature [14,15]. We did not consider gradual increase in Dab/Tra doses responsible for this delay. However, regarding our treatment failure in this case, we consider that undetermined genetic mutations beyond BRAF V600E might be involved, such as the PI3K/AKT/mTOR pathway or *CSF1R* genes reported in LCH and ECD [27]. At this time, we plan to continue the Dab/Tra regimen, expecting further gradual improvement of neurological deficits, since disease worsening has been observed upon interruption of targeted therapy [28,29].

Regarding AEs associated with Dab/Tra, previous reports showed grade 1/2 toxicities such as pyrexia (43%) and fatigue (28%) as the most common [30]. In a melanoma study, Amagai et al. found pyrexia, skin rash, and liver dysfunction [31]. Acute kidney injury was also noted in the use of the Dab/Tra regimen [32]. On the other hand, vemurafenib was noted to develop hepatic toxicity with AST/ALT elevation in 25% cases (grade 3/4 was noted in 0.5%) [33]. However, severe AEs like heart failure were rare [34]. In our case, treatment with Dab (max. 250 mg/day) and Tra (max. 2 mg/day) did not result in AEs like pyrexia and rash, except for grade 1/2 liver dysfunction with elevated alkaline phosphatase/choline esterase, and AST/ALT; however, no dose adjustments or interruption of treatment were required.

## 4. Conclusions

We report a case of CNS disease associated with mixed histiocytosis (LCH/ECD) treated with Dab/Tra six years after the onset of neurological symptoms, showing a delayed neurological response compared with previously reported cases. This delay was likely due to the late initiation of targeted therapy or the characteristic refractoriness to treatment in this case. Future considerations include the importance of rapidly differentiating histiocytic disorders in patients presenting with CLIPPERS-like CNS disease and introducing targeted therapy (BRAF/MEK inhibitors) at the earliest stage and at an optimal dosage. Long-term follow-up remains essential for all patients receiving targeted treatments.

## Figures and Tables

**Table 1:** Treatment response.

Time from Dab/Tra	Neurological Symptoms		CNS-MRI **	PET/CT *** (Maxilla-Mandibular Bones)	PET-CT (Long Bones)
	SARA Score	ICARS Score			
Onset of TT	21/40	33/100	active	PR	CMR
Dab (50-100-150-200)/Tra (0.5–1.5) *					
7 months	13/40	28/100	sl. improved	PR	pCMR
Dab (200)/Tra (1.5)					
18 months	14/40	29/100	NT	PR	pCMR
Dab (250)/Tra (2.0)					
21 months	17/40	31/100	sl. improved	PR	pCMR

Abbreviations. TT = targeted treatment; Dab = dabrafenib; Tra = trametinib; NT = not tested; PR = partial remission; CMR = complete metabolic response; pCMR = probable complete metabolic response. * A gradual increase in doses of Dab and Tra was described in the text. ** The pons lesion was read by the same radiologist. *** The bone lesions by the PET/CT were evaluated based on the Deauville scores (PR = DS 4/5, CMR = complete metabolic response (DS 1/2), pCMR = probable CMR; DS-3).

**Table 2:** Publications of vemurafenib treatment for CNS-ECD.

Authors [Ref]	Age/Sex	CNS Symptoms (Duration of CNS Symptoms to Dx of ECD)	Targeted Drugs	Duration of Vem (Months)	Outcome
Marano [11]	67/M	Chorea Ataxic gait	Vem	5	SD
Miron [9]	55/M	Chorea	Vem	NA	NA
Pan [8]	67/F	Ataxia CLIPPERS	Vem	NA	Response noted at 1 month; disease-free for 3 years
Bradshaw [7]	52/M	CLIPPERS (6 years)	Vem + St	10	Improved
Todisco [10]	52/M	Ataxic gait SARA 14/40	Vem	9	Improved SARA 9/40
Fernández-Eulate [12]	31/F	Unstable gait	Vem	10	PR; 3 years later SD
Mazor 1 [13]	51/F	(2 years)	Vem	52	Response noted at 3 months
Mazor 2 [13]	64/M	(8 years)	Vem	14	Response noted at 4 months
Mazor 3 [13]	43/M	(9 years)	Vem	6	Response noted at 1 month

Abbreviations: CLIPPERS = chronic lymphocytic inflammation with pontine perivascular enhancement responsive to steroids; SARA = Scale for the Assessment and Rating of Ataxia; Vem = vemurafenib; St = steroid; PR = partial response; SD = stable disease; NA = not available.

**Table 3:** Dabrafenib with or without trametinib in CNS-ECD cases.

Case No.	Age (Yrs.)/ Gender	Regimens (Max Doses/Day)	Regimen Starts from Dx	Improvement of Neurological Symptoms
Al Bayati [15]	44/F	Dab/Tra (300 mg/2 mg)	2 years	in 4 months
Yuen [14]	44/M	Dab (not reported)	7 weeks	in 3 months
Present case *	46/F	Dab/Tra (250 mg/2 mg)	6 years	in 7 months (transient)

Abbreviations: F = female; M = male; Dab = dabrafenib; Tra = trametinib). * Present case is not CNS-ECD, but CNS-mixed histiocytosis (LCH/ECD).

## Data Availability

The original contributions presented in this study are included in the article. Further inquiries can be directed to the corresponding author.

## Abbreviations

The following abbreviations are used in this manuscript:

ECD	Erdheim–Chester Disease
LCH	Langerhans cell histiocytosis
CNS	Central nervous system
CLIPPERS	chronic lymphocytic inflammation with pontine perivascular enhancement responsive to steroids
Dab	dabrafenib
Tra	trametinib
DS	Deauville score
